# After a catastrophe, a little bit of sex is better than nothing: Genetic consequences of a major earthquake on asexual and sexual populations

**DOI:** 10.1111/eva.12967

**Published:** 2020-04-22

**Authors:** Ronan Becheler, Marie‐Laure Guillemin, Solenn Stoeckel, Stéphane Mauger, Alice Saunier, Antonio Brante, Christophe Destombe, Myriam Valero

**Affiliations:** ^1^ Centro de Conservación Marina Departamento de Ecología Facultad de Ciencias Biológicas Pontificia Universidad Católica de Chile Casilla Chile; ^2^ UMI 3614 Evolutionary Biology and Ecology of Algae CNRS Sorbonne Université Universidad Austral de Chile Pontificia Universidad Católica de Chile Roscoff France; ^3^ Instituto de Ciencias Ambientales y Evolutivas Facultad de Ciencias Universidad Austral de Chile Valdivia Chile; ^4^ UMR1349 Institute for Genetics, Environment and Plant Protection INRA Le Rheu France; ^5^ Departamento de Ecología Facultad de Ciencias Universidad Católica de la Santísima Concepción (UCSC) Concepción Chile; ^6^ Centro de Investigación en Biodiversidad y Ambientes Sustentables (CIBAS) UCSC Concepción Chile

**Keywords:** aquaculture, conservation genetics, empirical population genetics, evolution of sex, habitat degradation

## Abstract

Catastrophic events can have profound effects on the demography of a population and consequently on genetic diversity. The dynamics of postcatastrophic recovery and the role of sexual versus asexual reproduction in buffering the effects of massive perturbations remain poorly understood, in part because the opportunity to document genetic diversity before and after such events is rare. Six natural (purely sexual) and seven cultivated (mainly clonal due to farming practices) populations of the red alga *Agarophyton chilense* were surveyed along the Chilean coast before, in the days after and 2 years after the 8.8 magnitude earthquake in 2010. The genetic diversity of sexual populations appeared sensitive to this massive perturbation, notably through the loss of rare alleles immediately after the earthquake. By 2012, the levels of diversity returned to those observed before the catastrophe, probably due to migration. In contrast, enhanced rates of clonality in cultivated populations conferred a surprising ability to buffer the instantaneous loss of diversity. After the earthquake, farmers increased the already high rate of clonality to maintain the few surviving beds, but most of them collapsed rapidly. Contrasting fates between sexual and clonal populations suggest that betting on strict clonality to sustain production is risky, probably because this extreme strategy hampered adaptation to the brutal environmental perturbation induced by the catastrophe.

## 1 INTRODUCTION

Catastrophic events, such as wildfires, storms, tsunamis, earthquakes, or volcanic eruptions, dramatically affect both population demography and genetic diversity (Lande, 1988, 1993), especially in sessile and sedentary organisms. The sharp reduction of the census size following these events generally leads to severe demographic bottlenecks and to subsequent impoverishment of genetic variation (Carson, 1990; Nei, Maruyama, & Chakraborty, 1975). Signatures of such bottlenecks result in the loss of allelic variants, especially the rarer ones, but the signal only persists a few generations after the catastrophic event (Luikart & Cornuet, 1998). In addition, the distribution of the remaining genetic diversity among individuals may also be modified, as reduced effective size increases the odds of mating among relatives (Frankham, 1998; Spielman, Brook, & Frankham, 2004). Such high stochasticity at both the genetic and demographic levels constitutes a fertile ground for shifts in reproductive mode. For example, in plants, a reduction of self‐incompatibility was observed in bottlenecked populations (Reinartz & Les, 1994) and increased rates of self‐fertilization are often detected at the retracting edges of a species' range (Levin, 2012; Pujol, Zhou, Sanchez Vilas, & Pannell, 2009).

Documenting the consequences of catastrophic events on genetic variation and reproductive system in the wild is difficult and relies on chance since they are, by nature, unpredictable. Indeed, most studies have focused on the consequences of a catastrophic event on genetic structure by analyzing the impacted populations a posteriori (Jacquemyn, Roldán‐Ruiz, & Honnay, 2010; Russello, Gladyshev, Miquelle, & Caccone, 2004) or sometimes benefitting from banks of ancient data (e.g., data from museum specimens: Nyström, Angerbjörn, & Dalén, 2006; archeological data: Hadly et al., 2004; Weber, Stewart, & Lehman, 2004). Population‐based studies in which information was gathered before and after the extreme event in order to quantify the changes in genetic diversity caused by the catastrophe are rare (but see Gallardo, Köhler, & Araneda, 1995; Hsu et al., 2017; Pujolar et al., 2011; Wilmer et al., 2011). These studies have reported a loss of genetic diversity by directly comparing the standing variation before and after the event. However, they all focused on obligatory sexually reproducing vertebrates, in particular on mammals (Bouzat et al., 1998; Gallardo et al., 1995; Groombridge, Bruford, Jones, & Nichols, 2001; Matocq & Villablanca, 2001; Wisely, Buskirk, Fleming, McDonald, & Ostrander, 2002) or fishes (Plath et al., 2010; Pujolar et al., 2011). If loss in genetic variation is expected regardless the rate of sex, the severity and the duration of the recovery phase should vary (Hörandl, 2006). As stated in Hörandl (2006), “sexuals can probably re‐establish genetic variation more effectively after a bottleneck (…), apomicts [i.e., asexuals or clonals], in contrast, need longer time periods and/or multiple colonizations for the creation of clonal diversity.” Indeed, if clonality could favor demographic resilience by maximizing reproductive insurance, the propagation of new alleles, originating from mutation or migration, is slowed down by the rarity of sex. On the other hand, large clonal lineages are more prone to survive severe demographic bottleneck. We thus hypothesized that clonality may buffer, at least to some extent, the loss of variation caused by extreme demographic reduction. Testing the hypotheses of Hörandl implies working along a gradient in the rate of clonality (corresponding to the relative frequency of the descendants resulting from clonal reproduction within a population; Marshall & Weir, 1979).

The red alga *Agarophyton chilense* (formerly *Gracilaria chilensis*) represents a good candidate to tackle these predictions about the evolutionary shifts caused by catastrophic events in partially clonal species. This species*,* as other species of Gracilariaceae (Krueger‐Hadfield et al., 2016), is able to shift between purely sexual reproduction (involving the alternation of haploid gametophytes and of morphologically similar diploid tetrasporophytes, both fixed to rocky substrate via holdfasts, forming fixed populations) and asexual propagation (via vegetative fragments of fronds capable of generating new free‐floating individuals growing on sandy/muddy bottoms; Guillemin et al., 2008). While asexual reproduction has never been reported in fixed populations of *A. chilense* (Guillemin et al., 2008; Guillemin, Valero, Faugeron, Nelson, & Destombe, 2014), humans have made use of this clonal ability for aquaculture development (Buschmann, Hernandez‐Gonzalez, & Varela, 2008). Indeed, *A. chilense* is one of the very few domesticated algae (Valero et al., 2017). Its farming began in the 1980s and relies on planting vegetative cuttings of both gametophytes and mostly tetrasporophytes (Guillemin et al., 2008). This recent anthropogenically induced high rate of clonality has already affected genetic and genotypic diversities of *A. chilense*. Notably, diploid heterozygous lineages may have been indirectly selected by farming practices (Guillemin et al., 2008). All information available about genetic diversity and reproductive system in *A. chilense* was gathered during sampling campaigns performed between 2004 and 2009 (Guillemin et al., 2008, 2014).

**FIGURE 1:**
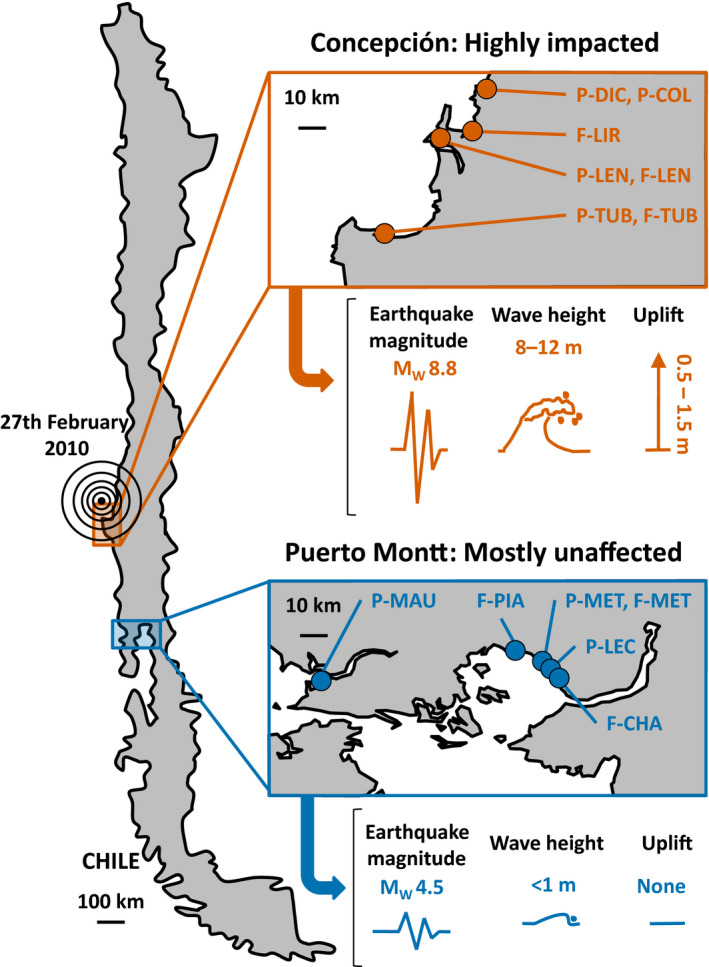
Sampling location of the six farmed populations (F), the seven natural populations (P) sampled in the region of Concepción, strongly impacted by the earthquake and tsunami of the 27^th^ February of 2010 and the region of Puerto Montt, mostly unaffected. Estimated magnitude of the earthquake (Richter scale) experienced in Concepción and Puerto Montt, height of the tsunami waves, and values of coseismic coastal uplift were taken from Castilla et al. (2010) and Vargas et al. (2011)

On the February 27, 2010, an earthquake of 8.8 magnitude (Richter scale) dramatically affected the coast of Central Chile (region of Concepción, Madariaga, Métois, Vigny, & Campos, 2010; see also Figure 1 hereafter). Coastal communities, including fishermen which were economically dependent on the farming and harvesting of *A. chilense*, were impacted by the huge waves of the resulting tsunami and the changes of coastal configuration (i.e., coastal uplift, Castilla, Manríquez, & Camaño, 2010). Ecosystems, housing, vessels, and infrastructure were heavily damaged in this region and led to permanent shifts in economic activities, including the desertion of algal farming in some of the previously most intensively harvested areas (Marín, Gelcich, & Castilla, 2014). Southward, the region of Puerto Montt was totally spared. These two regions benefited from a genetic survey starting before 2010 (see Guillemin et al., 2008) and were resampled immediately after the earthquake and tsunami of February 2010 and again 2 years later, in 2012.

These questions about the respective advantage of sex versus clonality in buffering the catastrophe and promoting population resilience, and the farmer's evolutionary role in this socio‐ecological system is central to develop conservation and management policies in *A. chilense.* They are also interesting for fundamental research on partially clonal organisms, fueling the current debate about genetic rescue (Whiteley, Fitzpatrick, ChrisFunk, & Tallmon, 2015; Willi, Kleunen, Dietrich, & Fischer, 2007).

The aim of this paper was to empirically address the predictions of Hörandl (2006) that sexuals should be able to restore genetic diversity more rapidly after a bottleneck than asexuals. Such studies are, to our knowledge, rare in primary producers and mostly nonexistent for partially clonal species. More specifically, we will test the following predictions: (a) The genetic variability is less impacted by the catastrophic event in farms, due to the enhanced buffering capacity of clonality; (b) the restoration of genetic variability is more rapid in sexual populations; and (c) putative shifts of reproductive strategies occurred in impacted populations. Finally, the current health status of natural and cultivated populations will be discussed in light of the impact of farmers have on their evolutionary trajectories.

## 2 MATERIAL AND METHODS

### 2.1 Model species and sampling strategy

*Agarophyton chilense* is characterized by a complex isomorphic life cycle with an alternation of independent haploid and diploid individuals (i.e., male and female gametophytes and tetrasporophytes, respectively) with a similar morphology. In the field, only fertilized female gametophytes are easily detected by eye. Distinction among nonmature individuals, reproductive but nonfertilized female gametophytes, male gametophytes, and tetrasporophytes is not possible. The sampling was therefore blind, and it was not possible to control, a priori, the number of diploid individuals collected in each site. The sex and stage (ploidy level) of the individuals was determined a posteriori by observations of the reproductive organs under a binocular microscope (Guillemin et al., 2008). For nonmature individuals, phase was determined using sex markers developed by Guillemin, Huanel, and Martínez (2012). Only diploid individuals were kept for analyses, for two main reasons*.* Diploids indeed represented the main portion of the samples, notably in farms (Guillemin et al., 2008). In addition, most of the methods of inference in population genetics were developed for diploids.

Temporal sampling of natural fixed populations (*N* = 7) and farms (*N* = 6) began in 2002 and was undertaken during the Austral summer months (i.e., January to March). Before the earthquake, sites were sampled at one or two time steps between 2002 and 2009 (Table 1). Sampling in 2010 was completed in March, <1 month after the earthquake. All sites were sampled in 2012.

**TABLE 1:** Sampling scheme over time. For each sampling site, the region of origin is provided, as well as its geographical coordinates

Label	Locality	Region	Type of populations	Geolocalization	*S*	Sampling dates							
						2002	2003	2004	‐‐‐	2009		2010	2012
DIC‐N	Dichato	Concepcion	Natural/Impacted‐EQ	36°32'48.41"S, 72°56'59.96"W	10	–	–	43		–	X	31	22
COL‐N	Coliumu	Concepcion	Natural/Impacted‐EQ	36°33'1.47"S, 72°57'17.05"W	10	–	–	–		18	X	31	31
LIR‐F	Lirquen	Concepcion	Cultivated/Impacted‐EQ	36°42'35.47"S, 72°58'38.67"W	100	–	–	–		16	X	14	18
LEN‐F	Lenga	Concepcion	Cultivated/Impacted‐EQ	36°45'50.69"S, 36°45'50.69"S	10e6	–	7	25		–	X	28	31
LEN‐N	Lenga	Concepcion	Natural/Impacted‐EQ	36°45'52.18"S, 73°10'30.39"W	1	–	34	19		–	X	X	X
TUB‐F	Tubul	Concepcion	Cultivated/Impacted‐EQ	37°13'51.40"S, 73°27'7.05"W	10e6	–	–	64		19	X	32	23
TUB‐N	Tubul	Concepcion	Natural/Impacted‐EQ	37°15'6.30"S, 73°26'33.28"W	1	–	–	28		10	X	X	X
MAU‐N	Maullin	Puerto Montt	Natural	41°36'59.38"S, 73°35'34.64"W	100	–	–	43		13	X	15	28
PIA‐F	Piedra Azul	Puerto Montt	Cultivated	41°30'23.58"S, 72°47'59.82"W	10e6	61	–	–		30	X	31	29
MET‐F	Metri	Puerto Montt	Cultivated	41°35'53.87"S, 72°42'19.38"W	100	–	–	28		–	X	25	28
MET‐N	Metri	Puerto Montt	Natural	41°35'53.31"S, 72°42'19.44"W	1	–	–	41		–	X	26	26
LEC‐N	Lenca	Puerto Montt	Natural	41°36'18.53"S, 72°41'32.03"W	100	–	–	–		19	X	31	19
CHA‐F	Chaica	Puerto Montt	Cultivated	41°37'27.01"S, 72°40'10.39"W	100	–	–	64		17	X	26	27

Note

In the column “type of populations,” we further indicate whether populations were *natural* (P) or *cultivated* (F) and whether they were impacted by the earthquake (in February 2010, gray column)—if so noted *Impacted*. A semiquantitative assessment of the population size (*S*) before the earthquake is provided, with 1 indicating a size a few meters square, 10 for few tens of m^2^, 100 for few hundreds of m^2^, and 10e6 for big farms covering many hectares. For each sampling date, the number of diploid samples used in this work is provided. The symbol “X” indicates that concerned populations disappeared after 2010.

Sampling was carried out in two Chilean regions where both *A. chilense* natural and farmed opulations are encountered: Concepción, heavily impacted by the earthquake of 2010 through both tsunami waves and coastal uplift (Castilla et al., 2010; Vargas et al., 2011), and Puerto Montt where this event went almost unnoticed (Figure 1).

### 2.2 DNA extractions, PCR amplifications, and locus scoring

DNA extractions followed the protocol recommended by Cohen et al. (2004). Due to the eventful evolutionary history of *A. chilense* (an initial founder event followed by recurrent demographic bottlenecks explained by overharvesting and domestication; Guillemin et al., 2014), only a few microsatellite markers have been shown to be variable in Chile (Guillemin, Destombe, Faugeron, Correa, & Valero, 2005). Indeed, previous studies showed that genetic diversity of *A. chilense* is highly reduced along the Chilean coast compared to the species region of origin (New Zealand) probably because of the combined effects of the founding event (that likely took place at the end of the Quaternary) and the recent human impacts of harvesting and cultivation practices (Guillemin et al., 2014). PCR amplification of five microsatellite loci and allele size scoring was performed according to Guillemin et al. (2005). For loci 2B2, 6C7, 7F12, and 8B2 (locus names follow Guillemin et al., 2005), PCR products were visualized on an ABI 3100 Sequencer fragment analyzer (Applied Biosystem). For the locus 7D3 (Guillemin et al., 2005), PCR products were run on 6.5% polyacrylamide denaturing gels in a LI‐COR DNA sequencer model 4200™ (LI‐COR). For all five loci, PCR products of five to ten individuals already genotyped for the study of Guillemin et al. (2008) were scored jointly with the new samples to ensure correspondence in allele size between temporal sampling. A second and third round of PCR and electrophoresis were performed for individuals with missing data.

Genotypes with missing data were not retained for the following analyses. Most genotypes, from sampling done before 2009, were already published in Guillemin et al. (2008). Temporal datasets for farms and wild *A. chilense* used in this study are available in DRYAD: https://doi.org/10.5061/dryad.9kd51c5d8.

### 2.3 Clonal and genetic diversities

Due to cultivation practices of *A. chilense*, the occurrence of clonal replicates is expected to be high in farms (Guillemin et al., 2008). The dataset was then first screened for identical multilocus genotypes (MLG). For each farm, the probability that identical MLGs originated from distinct reproductive events was assessed through the binomial probability P_sex_ (Arnaud‐Haond, Duarte, Alberto, & Serrao, 2007), estimated with Genclone v.2.0 (Arnaud‐Haond & Belkhir, 2007). We further used Mlgsim software v.2.0 (Stenberg, Lundmark, & Saura, 2003), as a complementary approach based on simulations, to assess whether identical MLGs could occur by chance given the allelic frequencies in the population (Halkett, Simon, & Balloux, 2005). Using the combination of these two approaches, we inferred the number of clonal lineages occurring in each farm. We then computed two complementary genotypic indices: (a) the clonal richness, estimated by *R* = (*G* − 1)/(*N* − 1), G being the number of clonal lineages, and *N* the number of sampled individuals and (b) the slope *β* of the Pareto distribution, an index of evenness of clonal size (Arnaud‐Haond et al., 2007). The higher the dominance of some clones, the lower is the slope *β* of the Pareto distribution.

Genetic analyses in farmed populations were then performed retaining a single copy of each clonal lineage. For both farmed and natural populations, gene diversity within locations was estimated through the mean number of alleles per locus standardized to the lowest number of sampling units (Â) and unbiased gene diversity (*H*_E_) (Nei, 1978) using Genetix v4.05 (Belkhir, Borsa, Chikhi, Raufaste, & Bonhomme, 2004). For each sampling site, the departure from panmixia was estimated through the *F* _is_ of Weir and Cockerham (1984) and its significance was assessed with a procedure of 1,000 permutations.

### 2.4 Estimation of rates of clonality in farms

The rate of clonality *c* was assessed using ClonEstiMate, an efficient and robust Bayesian method to infer rates of clonality from populations genotyped at two time steps (Becheler et al., 2017). We hypothesized that our populations of *A. chilense* evolve according to an extended Wright–Fisher model (hereafter noted eWFM) explicitly taking into account partial clonality (assuming stable population size, mutation rates, and reproductive mode; no migration and selection; equations available in Stoeckel & Masson, 2014). Based on the transition of genotypic frequencies between two time steps, ideally corresponding to two successive generations, ClonEstiMate allows inferring the most likely rate of clonality explaining such a transition. According to the recommendations provided by the authors, *c* was not calculated when the sampling dates were too distant (i.e., more than 2 years) as this time lapse probably involved more than two generations and could lead to underestimation of *c* (Becheler et al., 2017). Inferences were thus calculated between 2009 and 2010, and between 2010 and 2012, for each farm where data were available.

### 2.5 Data analysis

Two kinds of analysis were performed. First, the temporal behavior of population descriptors of clonal and genetic diversities was analyzed using the nonparametric descriptive boxplot method. Second, departures from the extended Wright–Fisher model were then tested. For both analyses, we distinguished natural from farmed populations and, for each kind of population, three grouping criteria were defined:

- Nonimpacted: This group encompassed the populations from the region of Puerto Montt (i.e., nonimpacted, all sampling years) and from the region of Concepción for sampling dates prior to the earthquake (years of 2002, 2003, 2004 and 2009).
- Impacted: This group encompassed the populations from the region of Concepción sampled in 2010 (i.e., just after the catastrophic event). Natural populations correspond to Dichato and Coliumo and farmed populations to Tubul, Lirquen, and Lenga (see Table 1).
- Recovery: This group encompassed the populations from the region of Concepción sampled in 2012, 2 years after the catastrophic event.

We first performed Mann–Whitney Wilcoxon's rank tests to detect any differences among groups in the distribution of each descriptor of diversity. We compared nonimpacted versus impacted groups and impacted versus recovery groups at the population scale, using indices of genetic diversity averaged over all loci per population. Given the low size of impacted and recovery groups for both natural populations and farms (*N* = 2 and 3, respectively), it is theoretically impossible to obtain *p*‐values of .05 or lower for some of the tests performed. For example, (a) when *N* _a_ = *N* _b_ = 2, the minimal *p*‐value, obtained if the group A is ranked as (1; 2) and the group B is ranked as (3; 4), is of 0.33; (b) when *N* _a_ = *N* _b_ = 3, the minimal *p*‐value, obtained if the group A is ranked as (1; 2; 3) and the group B is ranked as (4; 5; 6), is of 0.10. Theoretical minimal *p*‐values are provided with each boxplot. Despite the low statistical power expected for our dataset (few populations surveyed per group), this approach still provides a qualitative view of averaged genetic evolution observed between groups.

A second analysis was then performed at the locus scale, considering loci as segregating independently, a reasonable assumption in fully sexual populations and in partially asexual populations of large size (Navascués, Stoeckel, & Mariette, 2010; Stoeckel, Porro, & Arnaud‐Haond, 2019). However, in the case of farmed populations, in which rates of clonality are expected to be very high, this assumption could not stand especially if high levels of linkage disequilibrium are observed between loci (Guillemin et al., 2008).

The concept of effective size is hardly extendable to clonal populations, as it was developed for purely sexual organisms; therefore, it is not recommended to infer demography from genotypes in partially clonal populations. We thus propose an alternative methodology to evaluate the consequences of the catastrophic event on the genetic evolution of populations.

Transitions of genotypic frequencies measure the changes in frequencies of genotypes at one locus, in a population, from time *t* to *t* + 1. Assuming that populations fit with a mathematical population genetics model, with known and stable evolutionary forces (here, the eWFM described above), the probability of these transitions can be computed using CloNestiMate (Becheler et al., 2017). This approach aimed at testing whether the earthquake altered the evolutionary forces (e.g., an increased strength of drift due to strong demographic reduction).

Briefly, nonimpacted populations should fit the typical transitions of genotypic frequencies expected under stable evolutionary forces and constant size (with high transition probabilities). In contrast, impacted populations should exhibit transitions with lower probabilities: The lower the transition probabilities, the higher the departures from the eWFM.

Using the three groups described above, we aimed to test whether (a) transition probabilities estimated between 2009 and 2010 were lower in impacted populations than in nonimpacted populations and (b) transition probabilities of recovering populations (i.e., between 2010 and 2012) were higher than just after the earthquake (i.e., between 2009 and 2010).

For farms, where asexual reproduction has been reported (Guillemin et al., 2008), they were estimated for incremental values of *c* (the rate of clonal reproduction): 0, 0.1, 0.2, 0.3, 0.4, 0.5, 0.6, 0.7, 0.8, 0.9, 0.95, 0.98, 0.99, 0.999, and 1 (Becheler et al., 2017). For natural populations, maintained only by sexual reproduction and spore settlement (Guillemin et al., 2008), these probabilities were computed assuming a fixed value of *c* = 0. Mann–Whitney Wilcoxon's rank tests were then performed to compare the probabilities of transitions of the nonimpacted versus impacted groups and the impacted versus recovery groups.

## 3 RESULTS

### 3.1 Genetic diversity of natural sexual populations, before and after the earthquake

Among the seven natural populations sampled, genetic diversity was low, independent of sampling year (0.28 < *H*_E_ < 0.59 and 1.6 < Â < 3.4; Table 2). Most populations (75%) did not depart from Hardy–Weinberg equilibrium (HWE, nonsignificant *F* _is_ in 15 out of 20). Five *F* _is_ values were significantly different from 0: two of them were positive and three were negative (Table 2).

**TABLE 2:** Genetic diversity of natural populations of *Agarophyton chilense* from the regions of Concepción and Puerto Montt, per sampling date

Region	Sampling site	Year	Effect EQ	*H*_E_	Â	*F*_IS_
Concepcion (impacted by earthquake)	Dichato	2004	Before	0.37 (0.27)	2.8	0.16 (**)
		2010	EQ	0.28 (0.26)	1.6	0.01 (ns)
		2012	After	0.33 (0.23)	2.4	0.22 (*)
	Coliumo	2009	Before	0.31 (0.26)	2.0	0.03 (ns)
		2010	EQ	0.31 (0.28)	1.8	0.08 (ns)
		2012	After	0.35 (0.21)	2.4	0.14 (ns)
	Lenga	2003	Before	0.44 (0.16)	3.0	−0.20 (**)
		2004	Before	0.39 (0.16)	2.8	−0.02 (ns)
	Tubul	2004	Before	0.59 (0.11)	3.2	0.10 (ns)
		2009	Before	0.59 (0.12)	3.4	0.06 (ns)
Puerto Montt (nonimpacted)	Maullin	2004	Nonimpacted	0.46 (0.04)	2.6	−0.04 (ns)
		2009	Nonimpacted	0.50 (0.08)	2.6	−0.13 (ns)
		2010	Nonimpacted	0.56 (0.07)	2.6	0.04 (ns)
		2012	Nonimpacted	0.52 (0.08)	2.8	0.11 (ns)
	Metri	2004	Nonimpacted	0.30 (0.27)	1.8	−0.21 (*)
		2010	Nonimpacted	0.29 (0.27)	1.6	0.11 (ns)
		2012	Nonimpacted	0.34 (0.24)	2.4	−0.08 (ns)
	Lenca	2009	Nonimpacted	0.30 (0.28)	1.8	0.05 (ns)
		2010	Nonimpacted	0.31 (0.25)	1.8	−0.36 (***)
		2012	Nonimpacted	0.34 (0.23)	2.4	0.00 (ns)

Note

The status of each population is described in the column “effect EQ.” For impacted populations, we distinguished three substatus, before earthquake (status before), directly after it (status EQ), and 2 years after (status after). Expected heterozygosity (*H*_E_) associated with its standard deviation, allelic richness (Â), and inbreeding coefficient *F* _is_ was computed with Genetix v4.05 (ns: nonsignificantly different from 0, *: *p* < .05, **: *p* < .01 and ***: *p* < .001).

In the impacted region of Concepción, the two small populations of Tubul (P‐TUB) and Lenga (P‐LEN) Figure 1) did not survive the tsunami and coastal uplift caused by the earthquake, while the two slightly larger natural populations (P‐DIC and P‐COL) persisted (Table 1). Recovery dynamics were therefore only studied in these two surviving populations.

**FIGURE 2:**
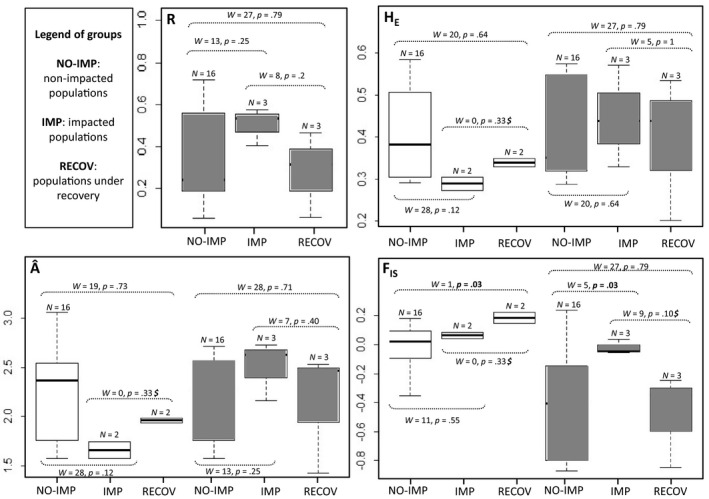
Comparisons of clonal richness (for the cultivated populations) and genetic diversity in groups of populations: populations mostly not impacted by the earthquake (NO‐IMP), populations sampled 1 month after being severely impacted by all the earthquake perturbations (IMP), and recovering populations sampled more than 2 years after the earthquake occurrence (RECOV). White boxes correspond to groups of natural populations and gray ones to farmed populations. The values of W, the statistic of the Mann–Whitney Wilcoxon's ranking test are provided

Changes in genetic diversity (*H*_E_), between sampling years, were generally small for these two sexual populations (Table 2). However, substantial changes just after the catastrophic event were detected in the region of Concepción with a decrease of 10% and 40% in allelic diversity (Â) in the populations P‐Col and P‐DIC, respectively. In contrast, in the nonimpacted region of Puerto Montt, the variations of Â were marginal, with an average decrease of only 4% in nonimpacted populations (Table 2). The lowest values of allelic richness in both populations of P‐DIC and P‐COL were detected in 2010. Results show that both *H*_E_ and Â (Figure 2) followed the same pattern with a decrease between the nonimpacted and impacted groups (Wilcoxon's rank test; population‐level: *p* = .12 for both *H*_E_ and Â; locus‐level:, *p* = .011 for Â and *W* = 277.5, *p* = .117 for *H*_E_) and then an increase between the impacted and recovery groups (Wilcoxon's rank test; population‐level: *p* = .33 corresponding to the minimal value when *N*_1_ = *N*_2_ = 2; locus‐level: *p* = .039 for Â and *p* = .425 for *H*_E_). This variation in diversity was corroborated by the loss of rare alleles in 2010. Among the seven alleles occurring at a frequency of 0.12 or lower in P‐DIC and P‐COL before the earthquake, only one was resampled in 2010. The recovery of the polymorphism is explained by the emergence of seven new rare alleles in 2012 samples (Table S1). In these impacted populations, the *F* _is_ increased between 2010 and 2012, from 0.01 to 0.22 in P‐DIC and from 0.08 to 0.14 in P‐COL (Table 2). These values were higher than the range of *F* _is_ observed in the nonimpacted group (Wilcoxon's rank test; population‐level: *p* = .030. locus‐level: *p* = .036; Figure 2), where variations of *F* _is_ were either positive or negative. Finally, departures from eWFM were greater in natural impacted populations than nonimpacted ones (Wilcoxon's rank test; population‐level: *p* = .029).

### 3.2 Clonal and genetic diversities of farmed populations, before and after impact

**FIGURE 3:**
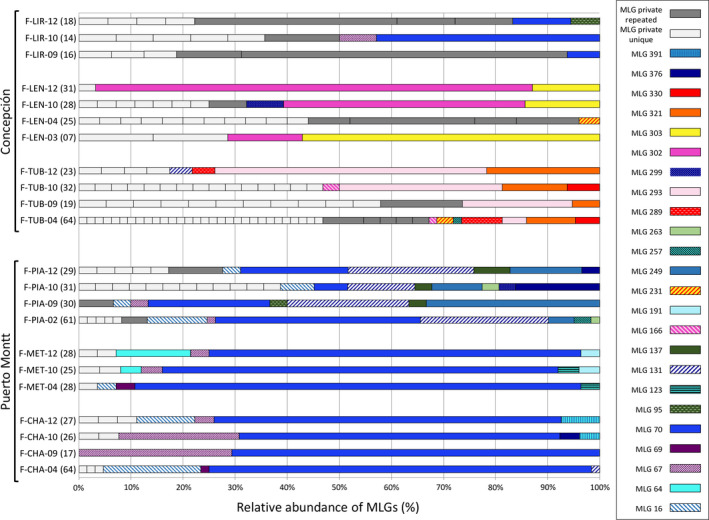
Relative abundance of shared and private multilocus genotypes (MLG) observed in farmed populations. The relative abundance is given over all the individuals genotyped in each farm for each sampling year. Private MLGs (unique or repeated) are represented using gray tones, while MLGs shared among farms and/or sampling dates are represented in colors. Labels used for farmed populations are the same as in Table 1. Numbers of sampled individuals are given in brackets

As expected, the farmed populations were highly clonal (Table 3), often dominated by one or few asexual lineages, as attested by low values of *R* and *β* (Table 3, Figures 2 and 3). The same MLGs were detected over the whole sampling period (from 2003 or 2004 to 2012; Figure 3). Most farmed populations exhibited negative *F* _is_ values, generally significantly different from 0 (*p* value <.05 for 15 of the 21 negative *F* _is_; Table 3). Inferred rates of clonality (*c*) varied between 0.2 and 1.

**TABLE 3:** Clonal and genetic diversities of the cultivated populations of *Agarophyton chilense*, from the regions of Concepción and Puerto Montt, per sampling date

Region	Sampling site	Year	Effect EQ	*c*		*N*_MLG_ (*N*_SU_)	*R*	β	*H*_E_	Â	*F*_IS_
Concepcion (impacted by earthquake)	Lirquen	2009	Before	0.8		6 (16)	0.33	>0	0.36 (0.21)	2.6	−0.63 ***
		2010	EQ		0.3	8 (14)	0.54	0.45	0.48 (0.30)	2.8	−0.06 (ns)
		2012	After			9 (18)	0.47	0.41	0.50 (0.11)	2.8	−0.36 ***
	Lenga	2003	Before	0.3		4 (7)	0.5	‐	0.44 (0.07)	2.2	−0.12 (ns)
		2004	Before			16 (25)	0.63	0.80	0.35 (0.20)	2.6	0.23 **
		2010	EQ		1	12 (28)	0.41	0.33	0.43 (0.19)	2.6	0.03 (ns)
		2012	After			3 (31)	0.07	0.05	0.24 (0.24)	1.6	−0.86 ***
	Tubul	2004	Before			42 (64)	0.65	1.15	0.59 (0.12)	3.0	−0.19 ***
		2009	Before	0.6		14 (19)	0.72	0.96	0.57 (0.11)	2.8	−0.01 (ns)
		2010	EQ		0.95	19 (32)	0.58	0.47	0.60 (0.12)	3.0	−0.05 (ns)
		2012	After			8 (23)	0.32	0.25	0.58 (0.12)	2.8	−0.26 **
Puerto Montt (nonimpacted)	Pietra Azul	2002	Nonimpacted			13 (61)	0.20	0.24	0.53 (0.10)	2.6	−0.32 ***
		2009	Nonimpacted	0.2		8 (30)	0.24	0.36	0.54 (0.15)	2.6	−0.45 ***
		2010	Nonimpacted		1	20 (31)	0.63	1.04	0.59 (0.09)	2.6	−0.08 (ns)
		2012	Nonimpacted			12 (29)	0.39	0.61	0.56 (0.14)	3.0	−0.35 ***
	Metri	2004	Nonimpacted			5 (28)	0.15	>0	0.33 (0.30)	2.0	−0.88 ***
		2010	Nonimpacted	0.95		7 (25)	0.25	>0	0.34 (0.25)	2.0	−0.82 ***
		2012	Nonimpacted			6 (28)	0.19	0.93	0.32 (0.23)	1.8	−0.81 ***
	Chaica	2004	Nonimpacted			7 (64)	0.10	0.07	0.46 (0.22)	3.0	−0.80 ***
		2009	Nonimpacted	1		2 (17)	0.06	>0	0.28 (0.26)	1.6	−0.85 ***
		2010	Nonimpacted		0.9	6 (26)	0.20	0.18	0.50 (0.17)	2.8	−0.38 ***
		2012	Nonimpacted			7 (27)	0.23	0.11	0.51 (0.18)	3.0	−0.5 ***

Note

The status of each population is described in the column “effect EQ” (effect earthquake). For impacted populations, we distinguished three substatus, before earthquake (status before), directly after it (status EQ), and 2 years after (status after). We estimated rates of clonality (*c* **)** from transitions of genotype frequencies between two sampling dates using ClonEstiMate. The number of multilocus genotypes (*N*_MLG_) over the number of sampling units (*N*_SU_), clonal diversity (*R*), and the parameter β of the Pareto's distribution was computed using Genclone v2.0. Expected heterozygosity (*H*_E_) associated with its standard deviation, allelic richness (Â), and inbreeding coefficient *F*_IS_ was estimated with Genetix v4.05 (ns: nonsignificantly different from 0, *: *p* < .05, **: *p* < .01 and ***: *p* < .001).

Aquaculture activities have now stopped in two farms (F‐TUB and F‐LIR) of the three studied in Concepción. In Tubul, a concession of 212 ha that was traditionally used for aquaculture since the 90s, a salt marsh crisscrossed by small channels of water, dried up almost completely after the earthquake due to coastal uplift (i.e., 1.6 m; Figure 1). Just after the earthquake, a few hectares of *A. chilense* farming beds still survived at the mouth of the estuary where both freshwater and saltwater influence exist (i.e., corresponding to our samples from 2010). In addition, most of the planting efforts that followed were directed to the creation of new beds directly on the sandy beach that faces the Pacific Ocean (i.e., corresponding to our samples from 2012). Of these new beds, only a few dying individuals were visible in 2013; all were dead in 2014.

Between 2010 and 2012, farms from both regions appeared highly clonal, with *c* ranging from 0.9 to 1. No clear pattern of variation was detected for *H*_E_ and Â among the nonimpacted, impacted, and recovery groups (Table 3 and Figure 2). In contrast, temporal variability in clonal composition (*R* and *β*) and departure from HWE (*F* _is_) were uncovered. Clonal diversity, and consequently excesses in heterozygosity, was quite stable in the nonimpacted farms, while large variations occurred in the region of Concepción after the earthquake (Table 3). The clonal richness decreased in all three impacted farms between 2010 and 2012 (i.e., impacted and recovery groups; Figure 2; Wilcoxon's rank test; population‐level: *p* = .2) and was logically accompanied by an increase in the dominance of a few clones (see decreasing β in Table 3 and Figure 3). *F* _is_ values were generally highly negative before 2010 in Concepción and in the farms of Puerto Montt whatever the sampling date (nonimpacted group). Just after the catastrophic event, they reached values close to 0 in the farms of Concepción (Wilcoxon's rank test; population‐level: *p* = .03; locus‐level: *p* = .001) and decreased again to significantly negative values in 2012 (Wilcoxon's rank test; population‐level: *p* = .10, corresponding to the minimal value when *N*_1_ = *N*_2_ = 3; locus‐level: *p* = .014) (Figure 2). In line with these increasing excesses of heterozygosity between 2010 and 2012, an increase in frequencies of few clonal lineages in the impacted farms of Tubul and Lenga was observed (i.e., F‐TUB and F‐LEN; Figure 3). These clonal lineages were already present at lower frequencies before the catastrophe. In the farm of Lirquen, the clonal composition seemed more deeply reshuffled (i.e., F‐LIR; Figure 3). Departure from the eWFM was more pronounced for farms impacted and under‐recovery than for nonimpacted farms (Wilcoxon's rank test; population‐level; between nonimpacted and impacted farms: *p* < .0001; between nonimpacted and under‐recovery farms: *p* = .172).

## 4 DISCUSSION

This study documents the genetic consequences of a major catastrophic event, an earthquake measuring 8.8 on the Richter scale, on sexual and partially clonal populations of seaweeds. Even with a background of low genetic polymorphism due to recurrent bottlenecks that had previously affected the species (both founder events related to colonization of the Chilean coast from New Zealand and intense harvesting for decades; Guillemin et al., 2008, 2014), populations of *A. chilense* bore the mark of the major earthquake of 2010. This event impacted both their demography and genetic diversity. Reproductive modes modulated earthquake consequences on genetic diversity both less than a month and still 2 years after the catastrophe. In this area, the farming of seaweed is an important social activity for local communities. It is based only or mostly on clonal fragmentation of thalli (Valero et al., 2017). The demographic and genetic consequences we observed, sometimes extreme such as the complete extirpation of local populations or the severe loss of alleles, may question the sustainability of this socio‐ecological system located along coasts prone to catastrophic events such as earthquakes and tsunamis. However, our results also showed that human practices in these farms played a role (while questionable from an evolutionary perspective) on population recovery and resilience.

### 4.1 Strong impact of the mega earthquake on natural populations and quick recovery due to migration

In the days following the earthquake, two natural populations from Concepción disappeared after the extreme modification of their habitat (N‐TUB and N‐LEN). These two small populations, of <1 m^2^ each, fixed to large boulders, were uplifted of 0.5–1.5 meters during the earthquake (Figure 1). Consequently, all established *A. chilense* individuals became completely emerged, quickly dried up and died off. This new topological context annihilated any chance of recolonization through recruitment. Complete eradication of upper intertidal populations of macroalgae (i.e., crustose coralline, kelp, and bulk‐kelp) has been previously reported after earthquakes (Chile: Castilla, 1988, Castilla et al., 2010; Vargas et al., 2011; Japan: Noda, Iwasaki, & Fukaya, 2016; New Zealand: Schiel et al., 2019). In Concepción, only two natural populations survived (N‐COL and N‐DIC). These two natural populations are located on gently sloping rocky platforms interlaced with deep crevices allowing the survival of individuals located in the low intertidal/high subtidal. Both N‐COL and N‐DIC showed a reduction of genetic diversity in 2010, just after the earthquake (Table 1, Figure 2), as expected in populations which sizes recently decreased (Carson, 1990; Luikart & Cornuet, 1998; Nei et al., 1975).

Surprisingly, genetic diversity in these two natural populations (estimated using allelic richness or expected heterozygosity) recovered to pre‐earthquake levels after only 2 years. Extreme mortality events have been shown to lead to a decrease in genetic diversity due to drift (Chan, Lacey, Pearson, & Hadly, 2005; Gallardo et al., 1995; Hsu et al., 2017; Pujolar et al., 2011; Reynolds, Waycott, & McGlathery, 2013). However, impacts of catastrophic events on population size and genetic diversity as well as the speed of rebound are highly idiosyncratic and depend on the magnitude of the disturbance, local geography (e.g., existence of barriers to gene flow), and species dispersal capacity (Brante et al., 2019; Hadly et al., 2004; Spiller, Losos, & Schoener, 1998). Less prolific species, or with lower dispersal capacity, may show more lasting effects of extreme mortality events on genetic diversity, with slow genetic recovery (Brante et al., 2019; Hadly et al., 2004; Wilmer et al., 2011).

The Gracilariales, as most macroalgae, have generally been considered as poor dispersers since their sexual free‐living stages are restricted to short‐lived gametes and spores that generally recruit in close proximity (few meters) to parents (Engel, Destombe, & Valero, 2004; Engel, Wattier, Destombe, & Valero, 1999). However, a recent work has shown that *A. chilense* is able to disperse over large distances and to quickly colonize novel habitats in low density‐blocking conditions (Guillemin et al., 2014). Effective dispersal and migration depend not only on the ability to emigrate from source populations, but also to become established into sink populations. This is often compromised in sessile organisms, such as algae, when the density of already established individuals is high (monopolization hypothesis, De Meester, Gómez, Okamura, & Schwenk, 2002). The quick genetic recovery observed in the two natural populations studied here may be explained by the fact that mass mortality due to the earthquake opened up space for migrant settlement. Our results argue for moderate‐to‐high connectivity of *A. chilense* in the Concepción region, in agreement with a previous study (Guillemin et al., 2014) that proposed that the species is capable of colonizing areas with few or no established conspecifics.

The two surviving natural populations departed more from the eWFM than nonimpacted ones during the short period of recovery, suggesting the violation of at least one of the underlying hypotheses of the model (constant population size and reproductive mode, and no “disturbing factor” in the sense of Wright, 1931 bringing gene frequencies similar to local ancestral ones). The assumption of “constant size” is the most likely violation, as the earthquake dramatically affected census and effective population sizes. This strong departure from the eWFM was not detected in 2010, immediately after the catastrophe since the signature in the genotypic transitions requires at least one round of reproduction. *A. chilense* recruits take months to grow and to mature (up to 2 years to reach their maximum size). At the sampling date of March 2010, the surviving individuals thus only represented a subset of the original population collected in 2009. Without at least one reproductive event, the demographic effect of the earthquake only mimicked a low sampling effort, explaining why the probabilities of transitions were slightly lower than in the nonimpacted group.

If the evolutionary trajectory of *A. chilense* natural populations appeared largely driven by the reduced effective size immediately after the earthquake, tracking the temporal dynamics of alleles can shed light on the role of migration during the recovery phase. Indeed, the majority of alleles occurring at low frequency (*c*. 0.10 and below) before the earthquake disappeared in 2010 (Table S1). A similar fate for rare and/or private alleles has been reported for the marble trout after flash flood events (Pujolar et al., 2011) and the colonial rodent tuco‐tuco following a volcanic eruption (Hsu et al., 2017). The resilience of the polymorphism is explained by the arrival of new rare alleles during the recovery, likely immigrating from neighboring populations where they occurred at higher frequencies (Table S1). This suggests that resilient populations functioned as open ones, violating a second hypothesis of the eWFM (i.e., absence of migration) as disturbing local gene frequencies (Wright, 1931). This is in line with former studies revealing an increased weight of the migration after disturbance that provide opportunities for recruitment (Becheler, Benkara, Moalic, Hily, & Arnaud‐Haond, 2014; Becheler, Diekmann, Hily, Moalic, & Arnaud‐Haond, 2010; Eriksson, 1993; Reusch, 2006). Increase in available open space lead, in some extreme cases, to mosaic patterns where impacted areas colonized by external sources are genetically distinct from the surviving local patches (Parvizi, Craw, & Waters, 2019) or to a reset of the genetic composition (Hsu et al., 2017; Wilmer et al., 2011) after the near‐total crash of populations. The surprising consequences of such profound genetic reset rely in a resulting diversity comparable to the original one, as the rapid recolonization is achieved through intense long‐distance migration (i.e., migration pool model), bringing new migrants and alleles in impacted populations. This phenomenon is highly similar to the reshuffling of genetic pools along axes of range expansion (Becheler et al., 2016; Bialozyt, Ziegenhagen, & Petit, 2006). The mixture of surviving local genotypes with immigrants from distant genetic pools could explain the increase of *F* _is_ in N‐COL and N‐DIC in 2010. Indeed, since the new alleles had not reached migration–drift equilibrium, this generates a spatial Wahlund effect.

The quick reestablishment of polymorphism in these sexual populations provides elements for optimism concerning the resilience of *A. chilensis* in the Concepción region in response to strong perturbations at short‐to‐medium time scale. Yet, drastic reductions in effective population size after catastrophe and during the recovery phase could lead to increased inbreeding (Frankham, 1998). *Agarophyton chilense* being a species presenting biphasic life cycle where independent haploid and diploid generations are encountered, the putative inbreeding depression could be mitigated by an effective purge of deleterious mutations during the haploid phase (Valero, Richerd, Perrot, & Destombe, 1992; but see Tortajada, Carmona, & Serra, 2009 or Szövényi et al., 2013). In this species, we could expect the deleterious effects of the transition phase (i.e., at most a few generations after the perturbation) would come to an end once the frequency of new migrant alleles rises in these affected populations.

### 4.2 High level of clonality in farms buffer immediate consequences of the catastrophe while hampering recovery

Despite the huge loss of cultivated areas caused by the tsunami (e.g., in F‐TUB; Marín et al., 2014), no loss of alleles was detected in farms and the clonal richness was only marginally reduced. This illustrates the immediate advantage of clonality, with disturbances affecting mostly the number of replicates of clonal lineages rather than the number of lineages itself, providing efficient buffering of the 2010 catastrophic event. The persisting genotypes could thus maintain an almost unaffected pool of alleles. However, the nature of the transition in genotypic frequencies departed from what we would expect under neutral evolution (revealed by the low probabilities of transition between 2009 and 2010 and the dynamics of *F* _is_). Again, this suggests the violation of at least one hypothesis underlying the eWFM in farms during this period, the more obvious being the hypothesis of constant size. Near‐complete mortality of *A. chilense* was registered only days after the earthquake in F‐TUB (Valdovinos, Muñoz, Sandoval, Vásquez, & Olmos, 2010) and the huge farm landings collapsed after the event (in F‐TUB in 2007–2009 landing was of *c*. 7,000 tons per year and of only 2 tons per year for the period 2013–2015, Rojas et al., 2017), supporting the idea of strong bottlenecks in farms located in the affected region of Concepción. Despite the persistence of repeated clones, the inbreeding coefficient lost its “clonal signature” (i.e., significant negative values) in Concepción farms sampled just after the earthquake. While *F* _is_ is expected to be highly negative in populations reproducing mostly asexually (Halkett et al., 2005), values raised up to almost zero, suggesting either a wider‐than‐local mixture of clonal lineages with less skewed allelic frequencies or a marked introgression of individuals coming from recent sexual events (Stoeckel et al., 2019) in impacted farms in 2010.

Short‐term evolutionary trajectories of genotypic diversity during the recovery phase (i.e., between 2010 and 2012) have proven highly informative and revealed subtle differences between farms probably linked with variation in amount and nature of the damage and postearthquake farmer's response. In the localities of Tubul and Lirquen (F‐TUB and F‐LIR), farmers lost practically everything after the destructive earthquake and tsunami's waves (houses, boats, and harvesting equipment; Contreras & Winckler, 2013; Marín et al., 2014). Lenga is located in a small bay somewhat protected from strong waves by the Hualpén and the Tumbes Peninsulas, and the F‐LEN farm and farmers' activities were not as affected by the earthquake and tsunami than in F‐TUB and F‐LIR (difference in mean landing between 2007–2009 and 2013–2015 was virtually nonexistent in F‐LEN with *c*. 20 tons per year whatever the period under study—data from S. Mesa Porcella—SUBPESCA). In F‐LEN, most of the algal production comes from the lower intertidal/high subtidal zone and is only extensively planted and harvested by local families. Farmers' response to the postcatastrophe socio‐ecological crisis splits into distinct scenarios: While activities in F‐LEN stayed somewhat unchanged, in F‐LIR the *A. chilense* farming was abandoned, while in F‐TUB farmers attempted to restock using survivors. In F‐LIR, only scattered thalli growing along the sandy banks that were previously farmed remained in 2012. An important reshuffling of clones was observed in F‐LIR (among the three clonal lineages detected in 2009 in our dataset, only one was recovered both in 2010 and 2012 while four new clones were sampled in 2012, see Figure 3). The massive importation of clones from distant populations (farmed or natural) by tsunami waves is consistent with the observed genetic patterns. On the contrary, the important increase of rate of clonality (*c*), reaching 100% between 2010 and 2012 in F‐TUB and F‐LEN, exemplifies the “planting from cuttings” strategy by farmers that reinforces the dominance of local surviving clones (especially MLG302 and MLG303 in F‐LEN and MLG293 and MLG321 in F‐TUB, see Figure 3). The heterozygosity of artificially propagated genotypes was higher than the average heterozygosity expected under HWE, explaining the drastic shift toward negative *F* _is_ values between 2010 and 2012. This shift may be explained as the expected result of either massive clonality under dominant genetic drift, heterosis, or mutation accumulation (Guillemin et al., 2008; Krueger‐Hadfield et al., 2016).

Indeed, in highly clonal populations, each possible genotype—heterozygous or homozygous—can be fixed by drift (Reichel, Masson, Malrieu, Arnaud‐Haond, & Stoeckel, 2016). Loci with multiple alleles, such as microsatellites, present more possible heterozygous than homozygous genotypes. Thus, heterozygosity tends to increase over time in highly clonal populations genotyped with such markers and evolving primarily under drift (Stoeckel et al., 2019). High heterozygosity could also be explained by heterosis, where clones having greater heterozygosity may have been indirectly selected by farmers because of higher survival or growth rate. Indeed, diploids show higher growth rate than haploids in *A. chilense* (Guillemin, Sepúlveda, Correa, & Destombe, 2013) possibly because of the presence of two alleles at specific loci. However, beyond genetic diversity, diploidy also provides an increased capacity for gene expression and better persistence of genetic signal along generations as compared to haploidy. Minimizing a possible effect of heterosis, a first study showed a lack of effect of heterozygosity on growth and physiological responses in Puerto Montt population during summer under nitrogen limiting conditions (Usandizaga, Camus, Kappes, Guillemin, & Buschmann, 2018). Finally, mutation accumulation during somatic division has also been reported in *A. chilense* farms (Guillemin et al., 2008). This simple mechanism could also increase heterozygosity of dominant clones without any effect of heterosis, especially in populations under environmental stress (Bruggeman, Debets, Wijngaarden, Visser, & Hoekstra, 2003).

Currently, aquaculture activities have stopped in F‐TUB and F‐LIR farms in the impacted area of Concepción. In Tubul, coastal uplift of 1.6 m had a much higher impact on *A. chilense* farming than the 8 m high tsunami wave and associated run‐up (Fritz et al., 2011; Marín et al., 2014; Melnick, Moreno, Motagh, Cisternas, & Wesson, 2012) as it dried up the 212 ha salt march traditionally used for aquaculture since the 90s. However, a few hectares of *A. chilense* farming beds still survived in 2010 at the mouth of the estuary where both freshwater and saltwater influence exist and new beds were created in 2012 directly on the sandy beach that faces the Pacific Ocean. Those two populations went extinct in 2014. While habitat loss is undoubtedly responsible for much of the lack of recovery of aquaculture activity in F‐TUB (Marín et al., 2014; Rojas et al., 2017), it is also possible that farmers from Tubul have generated a mismatch between historically selected genotypes growing in the salt marsh and the requirements of the newly planted habitats. A study realized in the Puerto Montt region showed that *A. chilense* monoclonal stands performed better than more genotypically diverse beds (Usandizaga et al., 2020), suggesting the existence of multi‐purpose genotypes (Baker, 1974; Lynch, 1984) in this species farms. Such super clones are expected to display a large plasticity, enabling them to cope with environmental fluctuations, and in some cases to be maintained over extensive time periods (Arnaud‐Haond et al., 2012; Reusch, Bostrom, & Stam, 1999). It seems that even if super clones populated F‐TUB, they were not able to cope with the drastic habitat change from a salt marsh to a sandy beach with direct oceanic influence. This evolutionary failure may be a first indication of the importance of the genetic background of *A. chilense* populations in farm resilience. This issue does not only concern the focal species of this study, but most of the cultivated red seaweed, as others species of Gracilariales and Gigartinales. Indeed, farms of *Kappaphycus* and *Eucheuma* are maintained mostly clonally with strict vegetative reproduction of a very few number of selected genotypes (Valero et al., 2017). Most of these types of clonal farms are regularly affected by catastrophic events such as typhoons (in the Philippines: Andriesse & Lee, 2017), earthquakes and tsunamis (in Japan and Chile: Kontar, Santiago‐Fandino, & Takahashi, 2016, present work). This work provides a clear example of the necessity for farmers to maintain adaptive potential in their cultivated populations. This is notably well known in several terrestrial clonal crops (e.g., cassava, *Manihot esculenta*). The traditional farming practices allow the integration of a few spontaneous genotypes coming from sexual reproduction in farmed and natural populations each year. Coupled with the weeding of plants germinated from seed presenting low growth rate, this strategy maintains high levels of genotypic diversity and high heterozygosity in these crops (Delêtre, Hodkinson, & McKey, 2017; Duputié, David, Debain, & McKey, 2007; Pujol, David, & McKey, 2005).

## 5 CONCLUSIONS

This case study reported distinct fates of sexual versus clonal populations after a natural disaster. This is attributable to differences in reproductive strategy and is in line with several theoretical expectations about the respective advantages of sex and clonality. While strict clonality (i.e., rate of clonality, *c*, close to 1) conferred a noticeable ability to buffer genetic impoverishment after a catastrophic event, it lowered the odds of survival at larger environmental and time scales. In contrast, populations reproducing sexually were more sensitive to the immediate genetic impacts of catastrophic events. Yet, genetic recoveries of these populations were fast, probably promoted by long‐distance migration and rapid integration, by sexual recombination, of new alleles into the surviving local genetic pool (Keller et al., 2001). Partial clonality could, however, allow combining the advantages of the two reproductive modes. Indeed, it was recently shown that trajectories of partially clonal species (i.e., for 0 < *c* < 0.9) were very similar to purely sexuals (Reichel et al., 2016; Stoeckel et al., 2019). This suggests that only a small amount of sex is sufficient to benefit from its evolutionary advantages after some generations.

Partial clonality is widely distributed within the tree of life, including ecologically central species (such as seagrasses, corals), many species of commercial or agricultural interest (tree‐species), or human parasites. The implications of the presented results and the recent theoretical developments in population genetics of partially clonal species go beyond the specific case of algal farming. Understanding the influence of this mixed reproductive mode on evolution is absolutely necessary for a world, where the frequency of dramatic fluctuations in population size is increasing. Notably, caricatural representations of clonality as a binary trait (i.e., purely sexual versus purely clonal) should no longer persist. On the contrary, future studies devoted to partially clonal species should consider the rate of clonality as a continuous variable and more fully appreciate its variation among populations and species, as well as its evolutionary influences. It is a prerequisite for relevant management plans of harvested species, conservation actions of threatened species, or efficient measures in public health. Finally, while even a small amount of sex is evolutionarily advantageous, clonality also constitutes an opportunity for successful genotypes to spread and persist, notably the *general purpose genotypes*. On this point, several questions remain unresolved. How do such genotypes arise? What are the evolutionary consequences of the presence, or introduction, of superclones into a population? It would be wise to resolve these questions prior to promoting *general purpose genotypes.*

## CONFLICT OF INTEREST

None declared.

## Supporting information

Table S1

## Data Availability

Data deposited in the Dryad repository: https://doi.org/10.5061/dryad.9kd51c5d8.
